# Which is the Best Laser for Treatment of Benign Prostatic Hyperplasia?

**DOI:** 10.1016/j.euros.2022.11.020

**Published:** 2022-12-21

**Authors:** Cesare Marco Scoffone, Cecilia Maria Cracco

**Affiliations:** Department of Urology, Cottolengo Hospital, Torino, Italy

Nomination of the best laser for endoscopic treatment of benign prostatic hyperplasia (BPH) is a very arduous task, deserving further specifications as well as open-minded and in-depth considerations, as unequivocal choice of a single laser as the best would be a rushed and shallow simplification.

According to the 2022 European Association of Urology guidelines [1], holmium:YAG (Ho:YAG), thulium:YAG (Tm:YAG), GreenLight, and diode lasers are the four types currently taken into consideration for BPH treatment. The recommendation is strong only for Ho:YAG enucleation of large prostates and GreenLight vaporization of medium-sized prostates. Recommendation is weak for any Tm:YAG procedure, and the evidence is insufficient for all other laser-based procedures. It should be noted that thulium fiber laser (TFL) is not even mentioned yet.

The present debate on the best option for BPH involves three lasers: the TFL “new kid on the block” [2] supported by Enikeev and Taratkin [3]; and two traditional lasers, Ho:YAG endorsed by Rodriguez Socarrás et al. [4] and Tm:YAG promoted by Herrmann et al. [5], both recently updated by substantial technological innovations [6], [7], [8]. It could be argued that exclusion of the GreenLight laser from the debate is somewhat unfair, but we believe that its robust but limited role in prostate vaporization in the era of endoscopic enucleation of the prostate (EEP) [1], [9] is sufficient as an exclusion criterion.

If “best” means “with the widest range of applications in BPH surgical management”, certainly the winner is the Tm:YAG laser. This continuous-wave laser has outstanding vaporization capability and excellent cutting properties, and is extremely versatile and suitable for prostate vaporization, vaporesection, vapoenucleation, and enucleation, with the latter also largely supported by mechanical preparation of the adenoma [1], [9].

However, we are now in the age of transurethral EEP [9], an acronym embracing any anatomic enucleating technique performed with a Ho:YAG, Tm:YAG, GreenLight, or diode laser, and recently also with TFL. If we consider EEP the best endoscopic treatment for BPH refractory to medication, the question automatically becomes “Which is the best laser for EEP?”, for which the answer is Ho:YAG, closely followed by TFL. Both these pulsed lasers produce bubbles for which the size, morphology, and sequence can be adjusted according to the laser settings (energy, frequency, pulse duration), laser peak power, pulse modulation, laser fiber diameter, and type of irrigation fluid, thus yielding different photomechanical effects [6], [7], [10], [11], [12]. The bubbles emitted by the fiber tip of a Ho:YAG laser are particularly effective in yielding real energy-driven (shockwave) dissection of an adenoma from the capsular plane [13], but the smaller TFL-induced bubble stream may also produce similar enucleating effects [12], with minimal use of mechanical detachment.

Considering the choice of laser in real-life clinical practice, the question may become more chameleon-like: “Which is the best laser for BPH/EEP in the hands of an individual surgeon, using different enucleation techniques, in an individual patient, in a specific department and hospital, in a specific country?”

An experienced surgeon might perform EEP using Ho:YAG with pulse modulation or Tm:YAG support for an almost blunt mechanical EEP without any problem, while a beginner could take advantage of the less aggressive TFL (slower, with less penetration depth and great hemostatic capabilities, and therefore a lower risk of intraoperative complications) [14].

A traditional three-lobe EEP (especially in a patient who has undergone repeated prostate biopsies or experienced recurrent urinary tract infections, with tight adherence between the adenoma and the capsular plane) might well benefit from a laser with excellent cutting properties such as Tm:YAG and TFL, whereas a totally en-bloc EEP may be well supported by a laser producing effective shockwave dissection, such as Ho:YAG used in no-touch mode, or TFL to a lesser extent. The surgeon’s personal preference for a specific laser also has to be taken into account (and this is usually a monogamous relationship) [15], [16].

The requirements of an academic department in a well-resourced hospital with a crowded emergency department in a large city will be completely different from those of a small urology unit in a suburban hospital with a lower number of patients and a limited budget. In the first case, more than one laser will be acquired, with alternatives to cover device malfunction or breakage. In the second case, the choice will fall on an effective and robust laser with versatility (applicable for BPH, urolithiasis, bladder and upper tract urothelial cancer, and ureteral and urethral strictures), easy assistance, and quick replacement. Up to 2021, Ho:YAG was the only versatile laser on the market able to treat the full array of pathologies mentioned [17], [18], but this modality has recently been joined by TFL [19], [20], [21], [22]. Pulsed Tm:YAG is now being applied in this clinical scenario [8], [9], [10], [11], with promise for overcoming the limitations of continuous-wave Tm:YAG lasers in stone treatment, but time is still not ripe to rank this laser at the same level as for Ho:YAG and TFL.

In low-resource countries, both Ho:YAG and Tm:YAG lasers might represent very demanding and delicate devices from both economic and customer assistance perspectives, as they require dedicated electrical plugs, a well-functioning refrigeration system, a stable electrical supply, and perfect alignment of the laser beam. By contrast, TFL devices would be much more suitable in this setting, as they are smaller, lighter, more resistant, and less demanding and expensive, with lower energy consumption.

In conclusion, we believe that the crux of the matter is not to unequivocally identify the best laser for BPH, but rather to be able to use any laser suitable for this purpose in the right way and in the right context, taking advantage of its potential, minimizing the risk of complications due to inappropriate intraoperative use, and tailoring the choice according to the patient’s clinical requirements and the local working environment. As usual, knowledge is key.

## Conflicts of interest

Cesare Marco Scoffone is a consultant for Ambu, Boston Scientific, Coloplast Porgés, Cook Medical, EMS, Karl Storz, Olympus, Promed, Quanta System, Rocamed, and Teleflex. Cecilia Maria Cracco is a consultant for Boston Scientific, Coloplast Porgés, EMS, Karl Storz, Olympus, Quanta System, and Teleflex.
